# Supplementary material for: Decisions bias future choices by modifying hippocampal associative memories
Source: Nat Commun. 2020 Jul 3;11:3318. doi: 10.1038/s41467-020-17192-7 (PMC7335207; doi:10.1038/s41467-020-17192-7)
Supplement: Supplementary file 1 — Supplementary Information [file 41467_2020_17192_MOESM1_ESM.pdf]

Supplementary Information

Decisions bias future choices by modifying hippocampal associative memories

Luettgau et al.

Supplementary Methods

For our proposed associative mechanism, we assumed that after Pavlovian conditioning, a CS would pre-activate the respective, associatively learned US and participants would make their decisions between CS based on the associated outcome. Our hypothesized mechanism relies on the assumption that participants form a (simple) model of the task, which is well in line with the literature on associative learning[1,2]. However, alternatively, the observed pattern of results could also be explained by a cached value account. According to this account, participants acquire cached values during Pavlovian conditioning and further use those model-free values to guide their decision, independent of associated outcomes or the learned relationships between CS and US. Our study was not designed to dissociate both mechanisms on a behavioral level. The prediction of the cached value account would be that decisions during choice-induced revaluation lead to changes in cached values and leave CS-US association unaffected. In other words, CS^0_A should have a reduced cached value, whereas the cached value of CS^+_A should be increased following choice-induced revaluation. In another fMRI contrast, we tested whether the relationship/similarity between CS^0_A and US^- (Supplementary Note 4) would change due to the choice-induced revaluation phase. If our results can be explained by the cached value account, we would assume that CS^0_A followed by US^- would show reduced activation/higher similarity in the post choice-induced revaluation run, compared to its equivalent partner stimulus CS^0_B followed by US^-. As any other stimulus- or outcome-related effects are controlled for in the contrast, and CS^0_A was not learned to be associated with US^- during Pavlovian conditioning (i.e. not being able to pre-activate a US^- representation), we assumed that reductions in activation could only be interpreted as a repetition of a shared feature, namely the stimulus/outcome value. After testing the contrast, we extracted parameter estimates from an independent hippocampus mask and correlated the parameter estimates with CP. We hypothesized that CS^0_A and US^- similarity would be negatively related to overall CP CS^0_A – overall CP CS^0_B. The lower the CS^0_A and US^- similarity, the less likely participants would be to select CS^0_A.

Supplementary Note 1

As an alternative to the proposed associative mechanism, parts of our functional neuroimaging results could also be explained based on cached values. During Pavlovian conditioning, participants might acquire incentive (cached) CS values and use these to guide their decisions, independent of CS-US associations. It is possible that revaluation choices changed these cached CS values, instead of the CS-US associations. Under this reasoning, one would assume that the value information conveyed by CS^0_A, the CS not chosen during revaluation and supposedly devalued, would become more similar to the value information of the low-valued US^- after choice-induced revaluation[3]. By its very nature, the fMRI-RS signal for learned CS-US associations represents BOLD signal reductions due to repetition of both value and identity features shared by CS and US, alongside the associative strength between CS and US[1]. However, CS^0_A should by design of the experiment not be capable to elicit any associative strength- or identity-related RS effects when followed by US^-, as it was never coupled with US^- during Pavlovian conditioning. Since value and identity of the US are inextricably linked in the present design, we reasoned that any RS signal changes for CS^0_A followed by US^- from PRE to POST would most likely be attributable to changes in valuation of CS^0_A. The cached value account would predict larger repetition suppression effects for CS^0_A followed by US^- as for CS^0_B followed by US^- during POST. Our design allowed us to set up a contrast to test this possibility (Supplementary Note 4). This indeed yielded effects in the right posterior hippocampus and a midbrain region in the vicinity of the dorsolateral substantia nigra pars compacta (Supplementary Figure 4) in POST. However, consistent with the absence of a behavioral revaluation effect for CS^0_A, the observed hippocampal effect did not differ PRE-POST (test on parameter estimates extracted from right hippocampal anatomical mask, Z = 0.90, P = .184, U_3 = .64, Wilcoxon signed-rank test, one-tailed). Importantly, the parameter estimates of the cached value effect in the right hippocampus and the associative effect in the left hippocampus were not correlated (all ρs < .18, Ps > .260, Spearman correlations, two-tailed). Even when extracting parameter estimates for cached value (Supplementary Note 4) and associative effect (Equation 1) from the exact same anatomical mask of the left

hippocampus, we did not observe significant correlations between the two contrasts (PRE: ρ = .22, P = .170; POST: ρ = –.03, P = .852; Spearman correlations, two-tailed), which would have been expected if both contrasts measure the same with flipped signs. According to the cached value account, hippocampal representations of CS^0_A-$US^−$ should be inversely related to preferences of CS^0_A. However, directly opposing this prediction, relationships of right hippocampus parameter estimates, and choice behavior were positive (all ρs < .31, P > 0.05, Spearman correlations, two-tailed), rendering an explanation of the observed behavioral results based on cached values unlikely. It should be noted that the cached value effects would have critically depended on choice-induced devaluation of CS^0_A. However, as we did not find behavioral support for the hypothesized devaluation effect, the cached value effects should be interpreted with caution.

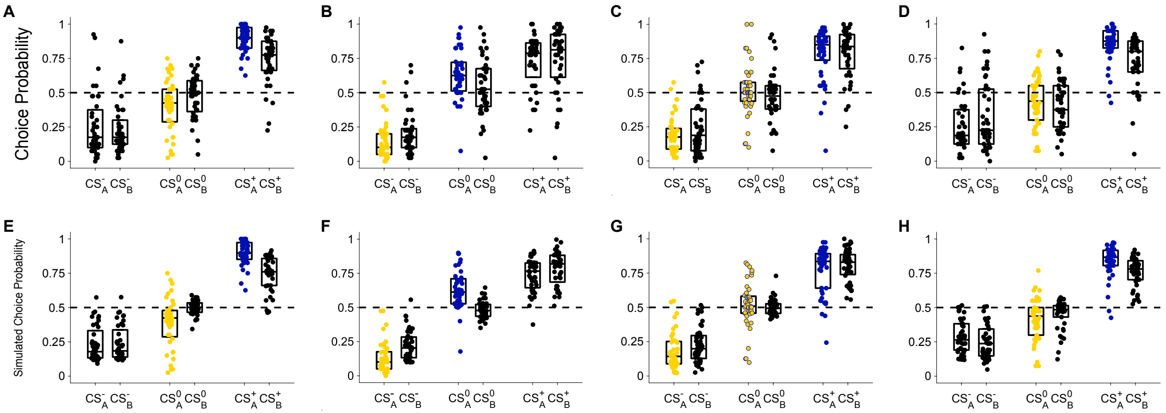

Supplementary Figure 1. Behavioral and simulation results.

A-D) Empirical choice results (as in Fig. 1E-H). Previously chosen CS (blue dots) are selected more often compared to equivalent CS (black dots) in Experiment 1 (A, N = 40, Z = 3.98, P < .001, Cohen's U_3 = .85, Wilcoxon signed-rank test, one-tailed), Experiment 2 (B, N = 40, Z = 2.20, P = .014, U_3 = .68, Wilcoxon signed-rank test, one-tailed), Experiment 5 (D, N = 42, Z = 3.03, P = .001, U_3 = .76, Wilcoxon signed-rank test, one-tailed) and previously unchosen CS (yellow dots) are selected less often compared to equivalent CS (black dots) in Experiment 1 (A, N = 40, Z = 1.97, P = .025, U_3 = .70, Wilcoxon signed-rank test, one-tailed) and Experiment 2 (B, N = 40, Z = 1.91, P = .028, U_3 = .66, Wilcoxon signed-rank test, one-tailed) during decision probe. The effect is not present in Experiment 3 (C, N = 44, Z = 0.41, P = .68, U_3 = .55, Wilcoxon signed-rank test, two-tailed), indicating that the roughly equal proportion of choices and non-choices of CS^0_A during revaluation had cancelled each other out. E-H) Averaged simulated choice probabilites (10,000 simulations per participant, Experiment 1: N = 40 (E), Experiment 2: N = 40 (F), Experiment 3: N = 44 (G), Experiment 5: N = 42 (H)), recapitulating observed empirical choice patterns. Please note that no statistical tests were performed on simulated data. Box plot center lines represent sample medians and box bottom/top edges show 25th/75th percentile of the (simulated) data, respectively. Source data are provided as a Source Data file.

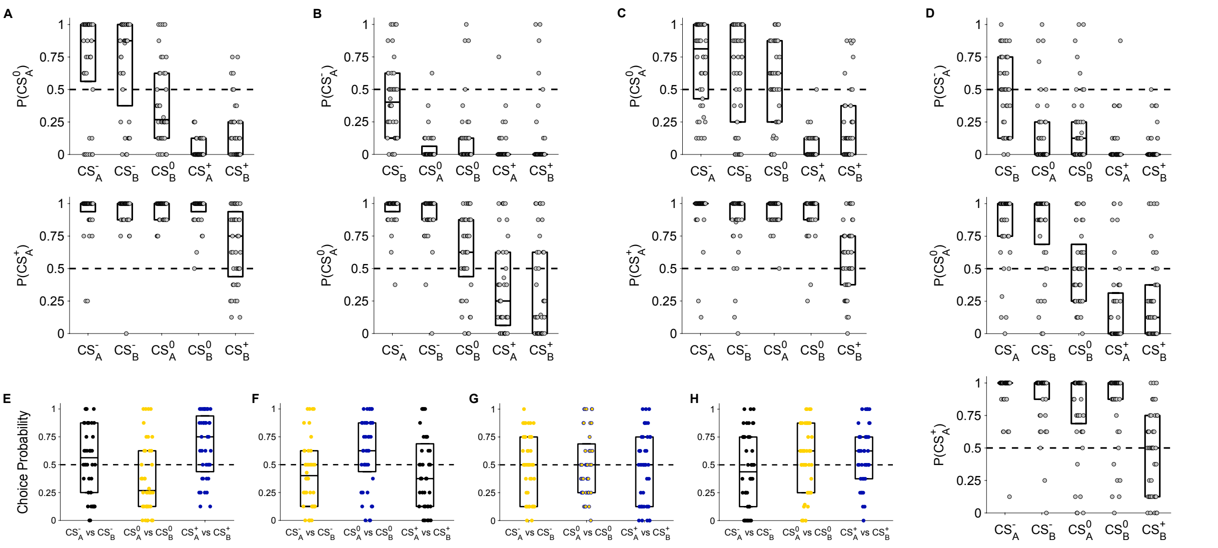

Supplementary Figure 2. Extended Behavioral Results and Pairwise Choice Probabilities.

Decision probe pairwise choice probabilities for CS that were presented during revaluation against every other CS. A) In Experiment 1 (N = 40), CS^0_A is only preferred over CS^-_A and CS^-_B (top), CS^+_A is the most preferred CS (bottom). B) In Experiment 2 (N = 40), CS^-_A is the least preferred CS (top), CS^0_A is chosen more frequently than CS^-_A, CS^-_B, and CS^0_B (bottom). C) In Experiment 5 (N = 42), CS^0_A is only preferred over CS^-_A and CS^-_B (top), CS^+_A is the most preferred CS (bottom). D) In Experiment 3 (N = 44), CS^0_A is chosen at the same frequency as CS^0_B (middle). Please note that since these plots serve descriptive purposes only, no statistical tests were performed. E-H) Pairwise within-category choice probabilities displaying the pairwise comparison that are most indicative of choice-induced preference changes. E) Experiment 1 (N = 40): CS^+_A and CS^+_B (Z = 3.43, P < .001, Cohen's $U3_1$ = .69, one-sample Wilcoxon signed-rank test vs. 0.5, one-tailed; CS^0_A and CS^0_B (Z = 2.05, P = .020, $U3_1$ = .68, one-sample Wilcoxon signed-rank test vs. 0.5, one-tailed), F) Experiment 2 (N = 40): CS^0_A and CS^0_B (Z = 1.93, P = .027, $U3_1$ = .68, one-sample Wilcoxon signed-rank test vs. 0.5, one-tailed); CS^-_A and CS^-_B (Z = 1.41, P = .079, $U3_1$ = .63, one-sample Wilcoxon signed-rank test vs. 0.5, one-tailed), G) Experiment 3 (N = 44): CS^0_A and CS^0_B (Z = 0.12, P = .905, $U3_1$ = .57, one-sample Wilcoxon signed-rank test vs. 0.5, two-tailed), H) Experiment 5 (N = 42): CS^+_A and CS^+_B (Z = 1.93, P = .027, $U3_1$ = .62, one-sample Wilcoxon signed-rank test vs. 0.5, one-tailed); CS^0_A and CS^0_B (Z = 1.07, P = .857, $U3_1$ = .62, one-sample Wilcoxon signed-rank test vs. 0.5, one-tailed). These results suggest that initially conditioned value was not overridden by revaluation choices and that the choice bias was mostly driven by the pairwise decisions of the respective revaluation CS against the same-value CS. However, repetitions of choices between the two revaluation CS also contributed to the observed overall choice probability effects. Box plot center lines represent sample medians and box bottom/top edges show $25^{th}/75^{th}$ percentile of the data, respectively. Source data are provided as a Source Data file.

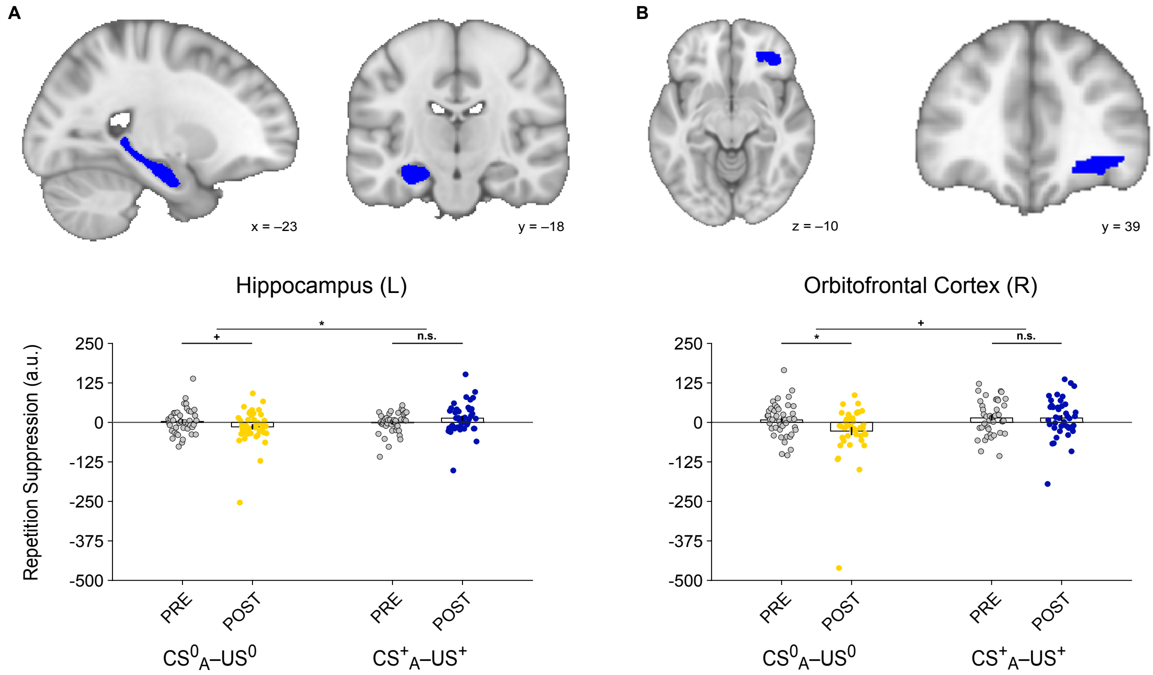

Supplementary Figure 3. Repetition suppression effects, separately for $CS^0{}_A$-US^0 and $CS^+{}_A$-US^+.

Extracted parameter estimates of the repetition suppression effects for $CS^0{}_A$-US^0, controlling for activation elicited by $CS^0{}_A$ followed by both incorrect outcomes (US^- and US^+) (Supplementary Note 2) and $CS^+{}_A$-US^+ repetition suppression, controlling for activation elicited by $CS^+{}_A$ followed by both incorrect outcomes (US^- and US^0) (Supplementary Note 3). A) Extracted parameter estimates from the left hippocampus (interaction effect: $F_{1, 41}$ = 4.31, P = .044, η^2_p = .10, 1–β = .99, rmANOVA). $CS^0{}_A$-US^0 showed a marginal effect from PRE to POST (Z = 1.82, P = .069, $U3_1$ = .64, Wilcoxon signed-rank test, two-tailed), but only a numerical difference was observed for PRE-POST change of $CS^+{}_A$-US^+ (Z = 1.56, P = .120, $U3_1$ = .69, Wilcoxon signed-rank test, two-tailed). B) Extracted parameter estimates from the right lateral orbitofrontal cortex (interaction effect: $F_{1, 41}$ = 3.51, P = .068, η^2_p = .08, 1–β = .99, rmANOVA). $CS^0{}_A$-US^0 showed a significant decrease from PRE to POST (Z = 2.44, P = .015, $U3_1$ = .71, Wilcoxon signed-rank test, two-tailed), but there was no evidence of a PRE-POST difference for $CS^+{}_A$-US^+ (Z = .01, P = .995, $U3_1$ = .52, Wilcoxon signed-rank test, two-tailed). Bar plots represent sample means. Error bars indicate standard errors of the mean. Asterisks indicate P-values < .05, plus signs represent P-values > .05 and < .10. Color bars indicate Z-values. Source data are provided as a Source Data file.

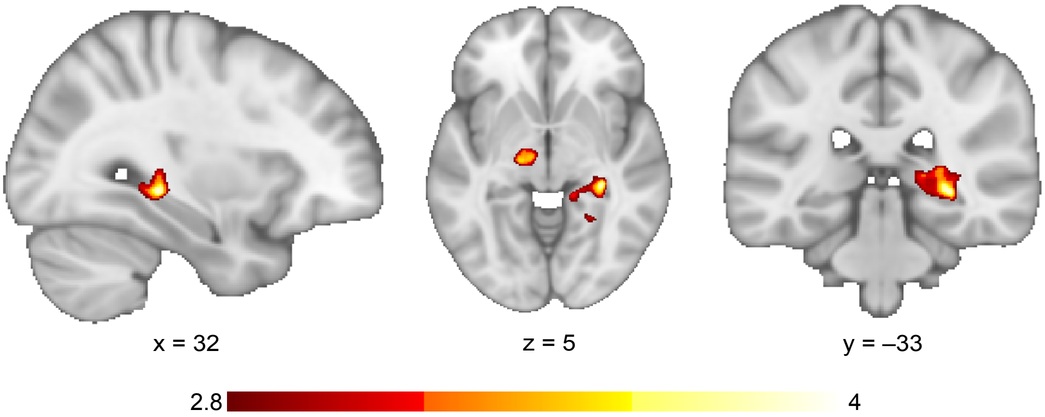

Supplementary Figure 4. Cached value control analysis results.

Whole-brain corrected pattern of activation (activation threshold $Z > 2.3$, cluster-forming threshold $P < 0.05$, thresholded at $Z > 2.8$ for display purposes) for the cached value control analysis (Supplementary Note 4) during the POST choice-induced revaluation fMRI run (N = 42). Two clusters in the right posterior hippocampus and in a left midbrain region in the vicinity of the dorsolateral substantia nigra pars compacta survived whole-brain correction. Extracting hippocampal activation with an independent anatomical right hippocampus mask and comparison of parameter estimates for PRE and POST yielded no significant difference (Z = 0.90, P = .184, U_3 = .64, Wilcoxon signed-rank test, one-tailed), suggesting no evidence for an influence of choice-induced revaluation decisions on activation. Additionally, we did not observe significant correlations between the cluster in the right posterior hippocampus from the cached value control analysis and the left hippocampus result from the associative analyses (all ρ*s* < .18, *Ps* > .260, Spearman correlations, two-tailed), suggesting different processes. Even when extracting parameter estimates for cached value (Supplementary Note 4) and associative effect (Equation 1) from the exact same anatomical mask of the left hippocampus, we did not observe significant correlations between the two contrasts (PRE: ρ = .22, P = .170; POST: ρ = –.03, P = .852; Spearman correlations, two-tailed), which would have been expected if both contrasts measure the same with flipped signs. Parameter estimates from the right posterior hippocampus were only marginally related with overall CP CS^0_A – overall CP CS^0_B: ρ_{40} = .30, P = .050 (two-tailed) and the within-category CP CS^0_A vs. CS^0_B: ρ_{40} = .29, P = .062 (two-tailed). However, the direction of the correlation was exactly opposite to the predictions of the cached value account. As there was no apriori anatomical hypothesis for the SNpc, we were unable to extract parameter estimates in an unbiased fashion. Thus, PRE and POST parameter estimates could not be compared. Left SNpc parameter estimates were not significantly related to left VTA parameter estimates from the associative analysis (all ρ*s* < .08, *Ps* > .63), nor with decision probe behavior (all ρ*s* < .20, *Ps* > .20). Color bar indicates *Z*-values.

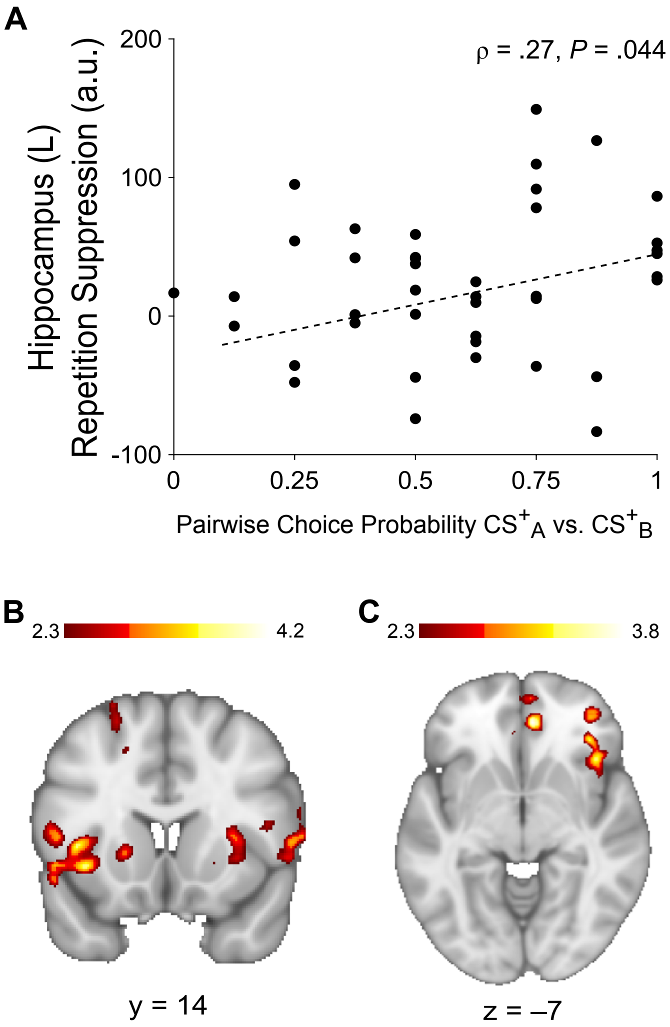

Supplementary Figure 5. Brain-behavior correlation and whole-brain regressions.

A) Left hippocampus parameter estimates (independent anatomical mask, Fig. 4A) for repetition suppression of CS^{+}_{A} relative to CS^{+}_{B} relative to CS^{+}_{B}-US^{+}, controlling for activation elicited by CS^{+}_{A} and CS^{+}_{B} followed by both incorrect outcomes (US^{-} and US^{0}) (Equation 2) during the POST choice-induced revaluation fMRI run positively correlate with decision probe pairwise choice probability of CS^{+}_{A} vs. CS^{+}_{B} (N = 42, ρ_{41} = .27, P = .044, Spearman correlation, one-tailed). The higher repetition suppression was after choice-induced revaluation, the more likely participants were to prefer CS^{+}_{A} over CS^{+}_{B}. B) CS^{+}_{A} preference vs. CS^{+}_{B} was correlated with POST choice-induced revaluation run repetition suppression (N = 42) in the bilateral anterior insula, suggesting choice-induced strengthening of CS-related pre-activation of neural ensembles coding for the used food items. C) We observed a positive relationship between PRE-POST changes in associative strength of CS^{0}_{A} and pairwise CP for CS^{0}_{A} vs. CS^{0}_{B}, in two clusters in the medial orbitofrontal cortex and lOFC, extending to the anterior insula (N = 42). Color bars indicate Z-values. Source data are provided as a Source Data file.

Supplementary Table 1. Computational Parameters and Model Fits

Exp.	Update	Model	$\alpha_{Learning}$	α_{ch}	α_{unch}	τ	*–LLE*	AIC_c
Exp. 1	RW	Model 1	< .001 (0 – 1)	-		.0002 (0 – 1.55)	63.43 (37.50 – 81.58)	133.59
		Model 2	.04 (0 – 1)	< .001 (0 – .78)	-	.20 (0 – 1.48)	40.52 (26.19 – 80.67)	98.10
		Model 3	.02 (0 – .58)	.50 (0 – .85)	.006 (0 – .86)	.15 (0 – .77)	37.27 (25.62 – 79.88)	**94.62**
	Asso. Value	Model 1	.002 (0 – 1)	-		.11 (.05 – 2.65)	41.35 (27.38 – 80.9)	97.10
		Model 2	.02 (0 – 1)	.002 (0 – .84)	-	.14 (.06 – 1.43)	40.60 (20.64 – 77.37)	95.87
		Model 3	.03 (0 – 1)	.42 (0 – .84)	.005 (0 – 1)	.17 (.06 – 3.37)	37.08 (20.60 – 81.88)	96.30
Exp. 2	RW	Model 1	.55 (0 – .95)	-		.45 (0 – 66.59)	53.41 (31.86 – 83.18)	115.99
		Model 2	.18 (0 – .72)	.005 (0 – .95)	-	.24 (0 – 3.44)	45.42 (15.43 – 82.02)	103.18
		Model 3	.04 (0 – .85)	.02 (0 – .88)	.005 (0 – .99)	.24 (0 – .77)	44.67 (5.55 – 77.11)	98.98
	Asso. Value	Model 1	.003 (0 – 1)	-		.15 (.05 – 2.65)	48.77 (15.81 – 83.23)	101.68
		Model 2	.04 (0 – 1)	.005 (0 – .92)	-	.20 (.002 – 6.11)	44.53 (15.71 – 82.81)	100.51
		Model 3	.03 (0 – 1)	.02 (0 – .99)	.005 (0 – .74)	.20 (.004 – 2.08)	44.38 (5.55 – 82.4)	**97.43**
Exp. 3	RW	Model 1	.03 (0 – .88)	-		.18 (0 – 1.55)	50.60 (22.15 – 82.66)	107.92
		Model 2	.02 (0 – .85)	< .001 (0 – .70)	-	.07 (0 – 16.92)	41.06 (14.50 – 82.99)	95.31
		Model 3	.01 (0 – .83)	.009 (0 – .55)	< .001 (0 – .40)	.08 (0 – 24.81)	40.47 (13.64 – 83.07)	93.15
	Asso. Value	Model 1	.005 (0 – 1)	-		.11 (0 – 1.44)	41.12 (13.65 – 82.64)	93.78
		Model 2	.02 (0 – 1)	.008 (0 – .76)	-	.16 (0 – 1.28)	40.29 (13.65 – 82.62)	93.84
		Model 3	.02 (0 – 1)	.012 (0 – .86)	.009 (0 – .80)	.13 (.004 – 2.01)	44.38 (5.55 – 82.4)	**91.96**
Exp. 5	RW	Model 1	< .001 (0 – 1)	-		< .001 (0 – 3.89)	71.61 (49.60 – 82.72)	143.94
		Model 2	< .001 (0 – .85)	< .001 (0 – 1)	-	.16 (0 – 5.05)	54.25 (22.54 – 82.64)	114.94
		Model 3	.04 (0 – .61)	.52 (0 – .85)	.008 (0 – .72)	.24 (0.03 – 1.02)	51.11 (22.54 – 81.86)	110.71
	Asso. Value	Model 1	.002 (0 – 1)	-		.16 (0.04 – 4.78)	55.11 (23.54 – 82.86)	112.97
		Model 2	.13 (0 – 1)	.007 (0 – 1)	-	.24 (0.05 – 4.81)	50.79 (23.37 – 82.61)	110.69
		Model 3	.18 (0 – 1)	.50 (0 – 1)	.009 (0 – .97)	.22 (.04 – 2.98)	49.25 (22.90 – 82.51)	**107.85**

Note: Median and range of computational parameters, negative log likelihood estimates (*–LLE*) and corrected Akaike Information Criteria (AICc) of the Rescorla-Wagner like (RW)[4] and Associative Value (Asso. Value) reinforcement learning models for the four experiments. Lowest AIC_c per experiment, which was considered to account best for the observed participants' choice data, penalized for model complexity and sample size (number of participants) in bold letters.

Supplementary Note 2

CS^0_A simple effect contrast:

$[\ 2 \times CS^0_A\text{-}US^0\ ] - [\ CS^0_A\text{-}US^- + CS^0_A\text{-}US^+\ ]$

Supplementary Note 3

CS^+_A simple effect contrast:

$[\ 2 \times CS^+_A\text{-}US^+\ ] - [\ CS^+_A\text{-}US^- + CS^+_A\text{-}US^0\ ]$

Supplementary Note 4

Cached value control analysis contrast:

$[\ 2 \times (CS^0_B\text{-}US^- - CS^0_A\text{-}US^-)\ ] - [\ (CS^0_A\text{-}US^0 - CS^0_B\text{-}US^0) - (CS^0_B\text{-}US^+ - CS^0_A\text{-}US^+)\ ]$

Supplementary References

1. Klein-Flugge, M. C., Barron, H. C., Brodersen, K. H., Dolan, R. J. & Behrens, T. E. J. Segregated Encoding of Reward-Identity and Stimulus-Reward Associations in Human Orbitofrontal Cortex. *J. Neurosci.* **33**, 3202–3211 (2013).
2. Barron, H. C., Dolan, R. J. & Behrens, T. E. J. Online evaluation of novel choices by simultaneous representation of multiple memories. *Nat. Neurosci.* **16**, 1492–1498 (2013).
3. Summerfield, C., Luyckx, F. & Sheahan, H. Structure learning and the posterior parietal cortex. *Prog. Neurobiol.* **184**, (2020).
4. Rescorla, R. A. & Wagner, A. R. A Theory of Pavlovian Conditioning: Variations in the Effectiveness of Reinforcement and Nonreinforcement. in *Classical conditioning II: current research and theory* (eds. Black, A. H. & Prokasy, W. F.) 64–99 (Appleton-Century-Crofts, 1972).
