# Supplementary material for: Decisions bias future choices by modifying hippocampal associative memories
Source: Nat Commun. 2020 Jul 3;11:3318. doi: 10.1038/s41467-020-17192-7 (PMC7335207; doi:10.1038/s41467-020-17192-7)
Supplement: Supplementary file 3 — Reporting Summary [file 41467_2020_17192_MOESM3_ESM.pdf]

natureresearch

Corresponding author(s): Lennart Luettgau

Last updated by author(s): May 19, 2020

Reporting Summary

Nature Research wishes to improve the reproducibility of the work that we publish. This form provides structure for consistency and transparency in reporting. For further information on Nature Research policies, see Authors & Referees and the Editorial Policy Checklist .

Statistics

For all statistical analyses, confirm that the following items are present in the figure legend, table legend, main text, or Methods section.

n/a	Confirmed	
☐	☒	The exact sample size (n) for each experimental group/condition, given as a discrete number and unit of measurement
☐	☒	A statement on whether measurements were taken from distinct samples or whether the same sample was measured repeatedly
☐	☒	The statistical test(s) used AND whether they are one- or two-sided<br>*Only common tests should be described solely by name; describe more complex techniques in the Methods section.*
☐	☒	A description of all covariates tested
☐	☒	A description of any assumptions or corrections, such as tests of normality and adjustment for multiple comparisons
☐	☒	A full description of the statistical parameters including central tendency (e.g. means) or other basic estimates (e.g. regression coefficient) AND variation (e.g. standard deviation) or associated estimates of uncertainty (e.g. confidence intervals)
☐	☒	For null hypothesis testing, the test statistic (e.g. F, t, r) with confidence intervals, effect sizes, degrees of freedom and P value noted<br>*Give P values as exact values whenever suitable.*
☒	☐	For Bayesian analysis, information on the choice of priors and Markov chain Monte Carlo settings
☒	☐	For hierarchical and complex designs, identification of the appropriate level for tests and full reporting of outcomes
☐	☒	Estimates of effect sizes (e.g. Cohen's d, Pearson's r), indicating how they were calculated

Our web collection on statistics for biologists contains articles on many of the points above.

Software and code

Policy information about availability of computer code

Data collection	Behavioral data was collected with custom experimental code running in MATLAB 2012b (MATLAB and Statistics Toolbox Release 2012b, The MathWorks, Inc., Natick, MA, USA, v8.0.0.783) & MATLAB 2019a (v9.6.0.1072779). fMRI data was acquired with a Siemens Prisma 3T MRI system (Erlangen, Germany).
Data analysis	Behavioral data and brain-behavioral correlational data was analyzed using custom analysis code running in MATLAB 2012b (v8.0.0.783), 2017a (v9.2.0.556344) & 2019a (v9.6.0.1072779) that is publicly available at GitHub (https://github.com/LLuettgau/revaluation) and the MATLAB-based Measures-of-Effect-Size-toolbox (https://www.github.com/hhentschke/measures-of-effect-size-toolbox, Retrieved January 10, 2020). fMRI data was analyzed using FMRIB Software Library (v6.0). Multivariate fMRI analyses were conducted using CoSMoMVPA, running in MATLAB 2017a (v9.2.0.556344). Power calculations and power analyses were performed using G*Power (v3.1.9.2).

For manuscripts utilizing custom algorithms or software that are central to the research but not yet described in published literature, software must be made available to editors/reviewers. We strongly encourage code deposition in a community repository (e.g. GitHub). See the Nature Research guidelines for submitting code & software for further information.

Data

Policy information about availability of data

All manuscripts must include a data availability statement. This statement should provide the following information, where applicable:

- Accession codes, unique identifiers, or web links for publicly available datasets
- A list of figures that have associated raw data
- A description of any restrictions on data availability

The raw behavioral data, univariate extracted parameter estimates, thresholded and unthresholded univariate Z-maps, neural pattern similarity correlation matrices and brain-behavioral correlation data that support the findings of this study are publicly available at GitHub (https://github.com/LLuettgau/revaluation). The neuroimaging raw data that support the findings of this study are available upon reasonable request from the corresponding author (LL). The neuroimaging raw

data are not publicly available due to them containing information that could compromise research participant privacy/consent. The source data underlying Figures 1E-I, 2D-E, 3A-B, 4B and Supplementary Figures 1A-H, 2A-H, 3A-B, and 5A are provided as a Source Data file. A reporting summary for this Article is available as a Supplementary Information file.

Field-specific reporting

Please select the one below that is the best fit for your research. If you are not sure, read the appropriate sections before making your selection.

☐ Life sciences ☒ Behavioural & social sciences ☐ Ecological, evolutionary & environmental sciences

For a reference copy of the document with all sections, see nature.com/documents/nr-reporting-summary-flat.pdf

Behavioural & social sciences study design

All studies must disclose on these points even when the disclosure is negative.

Study description

Various quantitative, cross-sectional experimental studies involving learning and decision making involving repeated-measures. fMRI study with repeated-measures of the same sample.

Research sample

Experiment 1: Otto-von-Guericke University Magdeburg students, 49 young, healthy volunteers (age: M = 23.93, SD = 2.9 years, 18 males)
Experiment 2: Otto-von-Guericke University Magdeburg students, 64 young, healthy volunteers (age: M = 23.47, SD = 3.79 years, 26 males)
Experiment 3: Otto-von-Guericke University Magdeburg students, 61 young, healthy volunteers (age: M = 23.26, SD = 3.27 years, 23 males)
Experiment 4: Heinrich Heine University Düsseldorf students, 52 young, healthy volunteers (age: M = 22.06, SD = 3.69 years, 20 males)
Experiment 5: Otto-von-Guericke University Magdeburg students, 58 young, healthy and magnetic resonance imaging (MRI)-compatible volunteers (age: M = 24.61, SD = 4.01 years, 30 males)

All samples are representative of healthy, young, highly-educated human subjects. Rationale: Investigate choice-induced revaluation effects in a healthy, young, neurologically normal population

Sampling strategy

Random sampling based on public advertisements. Sample sizes were determined based on power calculations assuming a small to medium effect size for main and interaction effects in a repeated-measures ANOVA (partial eta-squared ranging between .04 and .06), allowing for standard type I and type II error magnitudes.

Data collection

Data was acquired using pen and paper (sociodemographic information), computers (behavioral experiments) and an MRI system (functional neuroimaging data). Researcher were not blind to the experimental condition and study hypothesis during data collection. Lennart Luettgau acquired behavioral (supported by Halla Mulla-Osman and Nicola Harzen) and neuroimaging data (supported by Denise Scheermann and Halla Mulla-Osman)

Timing

Experiment 1, 2, 3, 5: July 14, 2017 - April 4, 2019; Experiment 4: January 7 - January 27 2020

Data exclusions

Experiment 1: Seven participants were excluded from statistical analyses due to lacking engagement in the cover task during the Pavlovian conditioning phase that served as an attentional control (<10 % responses in trials that required to indicate the color of the square surrounding the conditioned stimuli. Two additional participants had to be excluded due to not passing the manipulation check (high-value option selected < 50 % during choice-induced revaluation).
Experiment 2: Ten participants were excluded from statistical analyses due to lacking engagement in the cover task during the Pavlovian conditioning phase, and fourteen subjects were excluded due to not passing the manipulation check (intermediate valued option selected < 50 % during choice-induced revaluation), one additional participant had to be excluded due to a technical error.
Experiment 3: Ten participants were excluded from statistical analyses due to lacking engagement in the cover task during the Pavlovian conditioning phase, and six subjects were excluded due to not passing the manipulation check (high-value option selected < 50 % during choice-induced revaluation), one additional participant had to be excluded due to a technical error (no data was recorded).
Experiment 4: Twelve subjects were excluded from statistical analyses due to not passing the manipulation check (high-value option selected < 50 % during choice-induced revaluation).
Experiment 5: One participant fell asleep during the post-revaluation fMRI-repetition suppression (fMRI-RS) run, three participants discontinued the MRI acquisition (one due to claustrophobia, two reported a headache during task performance). Twelve additional subjects were excluded due to not passing the manipulation check (high-value option selected < 50 % during choice-induced revaluation).

Data exclusion criteria were not pre-established, but based on the following assumptions:
1) the hypothesized choice-induced revaluation effects could only be obtained if participants selected the high-value option >= 50 % during choice-induced revaluation.
2) the attentional control criterion was selected to ensure a baseline level of engagement of participants during the Pavlovian learning phase, which was necessary to establish CS-US associations.

Non-participation

One participant in experiment 5 dropped out/declined participation after reading the informed consent materials, as the participant only then realized that he had an important appointment within the next hour.

Randomization

As experiments were conducted in within-subjects designs, no randomization had to be performed.

Reporting for specific materials, systems and methods

We require information from authors about some types of materials, experimental systems and methods used in many studies. Here, indicate whether each material, system or method listed is relevant to your study. If you are not sure if a list item applies to your research, read the appropriate section before selecting a response.

Materials & experimental systems

n/a	Involved in the study
☒	☐ Antibodies
☒	☐ Eukaryotic cell lines
☒	☐ Palaeontology
☒	☐ Animals and other organisms
☐	☒ Human research participants
☒	☐ Clinical data

Methods

n/a	Involved in the study
☒	☐ ChIP-seq
☒	☐ Flow cytometry
☐	☒ MRI-based neuroimaging

Human research participants

Policy information about studies involving human research participants

Population characteristics	See above
Recruitment	Participants were recruited from the local student community of the Otto-von-Guericke University Magdeburg, and Heinrich Heine University Düsseldorf, Germany by public advertisements and via online announcements. Only participants indicating no history of psychiatric or neurological disorder and no regular intake of medication known to interact with the central nervous system were included.<br>Selection biases dependent on the recruitment method cannot be explicitly ruled out since participants themselves made the decision whether they wanted to participate and contact the experimenter based on the public announcements of the study. These biases are, if present, unlikely to affect the results since experiments were conducted in within-subjects designs (i.e. all participants experienced all conditions in each experiment) and hence no random assignment to conditions or experimental groups was needed. The investigated choice-induced revaluation effects can be considered general and not specific to a population of young, healthy and highly-educated individuals.
Ethics oversight	The study was approved by the local ethics committee at the medical faculty of the Otto-von-Guericke University Magdeburg, Germany (February 2nd 2018, reference number: 19/18)

Note that full information on the approval of the study protocol must also be provided in the manuscript.

Magnetic resonance imaging

Experimental design

Design type	Task-based event-related design
Design specifications	Two blocks of fMRI, containing each 360 trial (720 trials total), trial length: 1800 ms, intertrial interval: 2000-6000 ms (drawn from a discretized γ-distribution, shape = 2.01, scale = 1)
Behavioral performance measures	Correct responses during attentional control task. After a pseudo-random 20 % of trials, participants were presented with probe trials in which they were asked to indicate whether or not the previously seen CS-US-association matched the true CS-US-association learned during Pavlovian conditioning via button presses with their right and left index fingers on an MRI-compatible response box. Correct responses were rewarded with 0.05 € and incorrect responses or time-out trials (without a response by the participant within 2500 ms after onset of the probe trial) resulted in a 0.05 € penalty which would be summed up as a bonus upon completion of the experiment. Vverall probability of correct answers, excluding time-out trials: M = .92, SD = .06.

Acquisition

Imaging type(s)	Functional
Field strength	3 Tesla
Sequence & imaging parameters	Multi-band accelerated T2*-weighted echo-planar imaging (EPI) sequence (multi-band acceleration factor 2, repetition time (TR) = 2000 ms, echo time (TE) = 30 ms, flip angle = 80°, field of view (FoV) = 220 mm, voxel size = 2.2 × 2.2 × 2.2 mm, no gap). Per volume, 66 slices covering the whole brain, tilted by approximately 15° in z-direction relative to the anterior–posterior commissure plane were acquired in interleaved order.
Area of acquisition	Whole brain scan

Diffusion MRI ☐ Used ☒ Not used

Preprocessing

Preprocessing software	All fMRI analyses steps were performed using tools from the Functional Magnetic Resonance Imaging of the Brain (FMRIB) Software Library (FSL, v6.0). Preprocessing included motion correction using rigid-body registration to the central volume of the functional time series, correction for geometric distortions using the field maps and an n-dimensional phase-unwrapping algorithm (B0 unwarping), slice timing correction using Hanning windowed sinc interpolation, high-pass filtering using a Gaussian-weighted lines filter of 1/100 Hz. Functional data were spatially smoothed using a Gaussian filter with 6 mm full-width at half maximum (no spatial smoothing performed for multivariate data analyses).
Normalization	EPI images were registered with the high-resolution brain images and normalized into standard (MNI) space using affine registration linear (boundary-based registration) as well as nonlinear registration.
Normalization template	MNI152_T1_1mm
Noise and artifact removal	We applied a conservative independent components analysis (ICA) to identify and remove obvious artefacts. Independent components were manually classified as signal or noise based on published guidelines, and noise components were removed from the functional time series.
Volume censoring	No volume censoring was applied

Statistical modeling & inference

Model type and settings

Mass univariate, General linear models (GLMs) were fitted into prewhitened data space to account for local autocorrelations. The individual level (first level) GLM design matrix per run and participant included fifty box-car regressors in total. Thirty-six regressors coded for onsets and durations of all eighteen presented CS-US-association trials (each modelled as single events of 1800 ms duration), two regressors coded for onsets and durations of the three within-run pauses (each 45 s), two regressors coded for onsets and durations of the attentional control task probe, four regressors coded onsets and durations of left and right button presses (delta stick functions on the recorded time of response button presses) and the six volume-to-volume motion parameters from motion correction during preprocessing were entered. Regressors were convolved with a hemodynamic response function (γ-function, mean lag = 6 s, SD = 3 s). Two separate pre and post choice-induced revaluation second level (group level) GLMs were carried out by submitting individual level parameter estimates to mixed-effects statistics and ordinary least squares (OLS) regression for higher-level COPE estimation.

Effect(s) tested

1.) CS0A fMRI-RS contrast:

[2 x (CS0A-US0 – CS0B-US0)] – [(CS0A-US– – CS0B-US–) + (CS0A-US+ – CS0B-US+)]

2.) CS+A fMRI-RS contrast:

[2 x (CS+A-US+ – CS+B-US+)] – [(CS+A-US– – CS+B-US–) + (CS+A-US0 – CS+B-US0)]

3.) Conjunction fMRI-RS contrast:

[2 x (CS0A-US0 – CS0B-US0)] – [(CS0A-US– – CS0B-US–) + (CS0A-US+ – CS0B-US+)]
AND
[2 x (CS+A-US+ – CS+B-US+)] – [(CS+A-US– – CS+B-US–) + (CS+A-US0 – CS+B-US0)] (3)

4.) CS+A simple effect contrast:

[2 x CS+A-US+] – [CS+A-US– + CS+A-US0]

5.) CS0A simple effect contrast:

[2 x CS0A-US0] – [CS0A-US– + CS0A-US+]

6.) Cached value control analysis fMRI-RS contrast:

[2 x (CS0B-US– – CS0A-US–)] – [(CS0A-US0 – CS0B-US0) – (CS0B-US+ – CS0A-US+)]

Specify type of analysis: ☐ Whole brain ☐ ROI-based ☒ Both

Anatomical location(s)

Apriori, literature-bases and theory-driven hypotheses: Lateral orbitofrontal cortex (lOFC) and Hippocampus

An independent functional mask of a contrast investigating stimulus-outcome-associations from a previous study (Jocham et al., 2016, Neuron) (restricted along the z-direction from –6 to –14 to constrain spatial extent), was used for small-volume correction of the bilateral lOFC. The small-volume corrected

functional activation mask from the conjunction contrast was used to extract contrast parameter estimates of the CS0A contrast and the CS+A contrast. Additionally, an independent anatomical mask of the hippocampus (Harvard-Oxford Atlas) was used to extract pre and post choice-induced revaluation contrast parameter estimates of the CS0A contrast and the CS+A contrast for comparison of parameter estimates and brain-behavioral correlations.

Statistic type for inference
(See Eklund et al. 2016)

Cluster-based correction with an activation threshold of $Z > 2.3$ using a cluster-extent threshold of $P < .05$ was applied at the whole-brain level and in predefined regions of interest.

Correction

FWE

Models & analysis

n/a	Involved in the study
☒	☐ Functional and/or effective connectivity
☒	☐ Graph analysis
☐	☒ Multivariate modeling or predictive analysis

Multivariate modeling and predictive analysis

Multivariate pattern similiarity analysis (variant of representational similarity analysis, RSA) on fMRI data, 1−Pearson’s product-moment correlation coefficient (1−r) was calculated as a measure of pairwise dissimilarity between multivariate neural patterns of interest (all pairs of CS0A/CS0B and CS+A /CS+B, followed by the respective incorrect associates, i.e. US that had not been paired during Pavlovian conditioning), separately for PRE and POST and the two ROIs. Within-subject pairwise neural dissimilarity was subtracted from 1 (to create a measure of neural pattern similarity) and Fisher-Z transformed to closer approximate normally distributed data. We then calculated the within-subject PRE-POST change between the resulting pairwise neural pattern similarity measures (POST r – PRE r, Δ Pearson’s r).
